# Amphidromous but endemic: Population connectivity of *Rhinogobius gigas* (Teleostei: Gobioidei)

**DOI:** 10.1371/journal.pone.0246406

**Published:** 2021-02-11

**Authors:** Te-Yu Liao, Pei-Luen Lu, Yuan-Huan Yu, Wen-Chien Huang, Jen-Chieh Shiao, Hung-Du Lin, Wei-Cheng Jhuang, Tak-Kei Chou, Fan Li

**Affiliations:** 1 Department of Oceanography, National Sun Yat-sen University, Kaohsiung, Taiwan; 2 Department of Life Science, National Taitung University, Taitung, Taiwan; 3 Doctoral Degree Program in Marine Biotechnology, National Sun Yat-sen University, Kaohsiung, Taiwan; 4 Doctoral Degree Program in Marine Biotechnology, Academia Sinica, Taipei, Taiwan; 5 Institute of Oceanography, National Taiwan University, Taipei, Taiwan; 6 Affiliated School of National Tainan First Senior High School, Tainan, Taiwan; Southwest University, CHINA

## Abstract

*Rhinogobius gigas* is an amphidromous fish endemic to eastern Taiwan. Fishes with the diadromous behavior are expected to have a broader distribution range and higher genetic homogeneity despite that some amphidromous fishes with limited distribution are observed and *R*. *gigas* is an additional exception with a limited distribution range. *Rhinogobius gigas* has been documented to be retained inshore near the river plume with a short pelagic larval duration of 30–40 days, which may account for the endemism of this species. The short marine larval stage of *R*. *gigas* may imply a population genetic structure and the aim of the present study is to test whether the population genetic structure is present in *R*. *gigas*. To test the population genetic structure, fragments of mitochondrial displacement loop and cytochrome c oxidase subunit I were sequenced to provide molecular inference for genetic structure among populations. Sixty-nine haplotypes were identified among 191 *R*. *gigas* from 10 populations of eastern Taiwan and the mean haplotype and nucleotide diversities for all samples were 0.956 and 0.0024, respectively, implying a bottleneck followed by a recent population expansion further supported by Fu’s *Fs* (-26.6; *p* < 0.001) and Tajima’s *D* (-1.5; *p* = 0.037) values. The phylogenetic analysis revealed lack of genetic structure and the bush-like median joining network without commonly shared haplotypes supports the same scenario. The genetic homogeneity is probably due to the amphidromous life history providing the opportunity for passive larval transportation among the rivers through coastal currents in eastern Taiwan. The endemism to eastern Taiwan may be a consequence of complicated interactions among short pelagic larval duration, interspecific competition and coastal currents.

## Introduction

Amphidromy is a life history of diadromous organisms, in which adults inhabit fresh water and their pelagic eggs or larvae drifting into estuaries or open oceans before settling down [1]. Most amphidromous fishes are found in the tropical and subtropical areas and their planktonic larvae are considered to facilitate distant dispersals and population connections [2]. Indeed, some narrowly endemic gobies do reveal relatively shorter pelagic larval durations (PLD) compared to their widespread congeners. For instance, *Sicyopterus aiensis*, *S*. *sarasini*, and *Lentipes concolor* have PLDs of c. 80 days and show no genetic structure across their distribution range of Vanuatu, New Caledonia, and the Hawaiian Islands, respectively, while *S*. *lagocephalus* has PLD of c. 130–260 days and is distributed across the Indo-West Pacific although deep genetic divergence was observed between oceanic basins [3–5]. However, more and more studies focusing on amphidromous fishes showed that the PLD is not always a key factor in shaping the distribution range and population structure. Environmental changes like sea level fluctuations during glacial periods, formations of new islands, and even the behavior of larvae can cause dramatic effects [6–8]. On the contrary, even with a short PLD of c. 40 days, *Kuhlia rupestris* can colonize remote islands and extend its range throughout the Indo-West Pacific at an evolutionary timescale although the speciation may be ongoing [9,10]. In the light of the variety and complexity of amphidromous life history, case studies are important and could provide us better understandings of the population connection and evolution history of the mysteriously amphidromous fishes.

**Fig 1:**
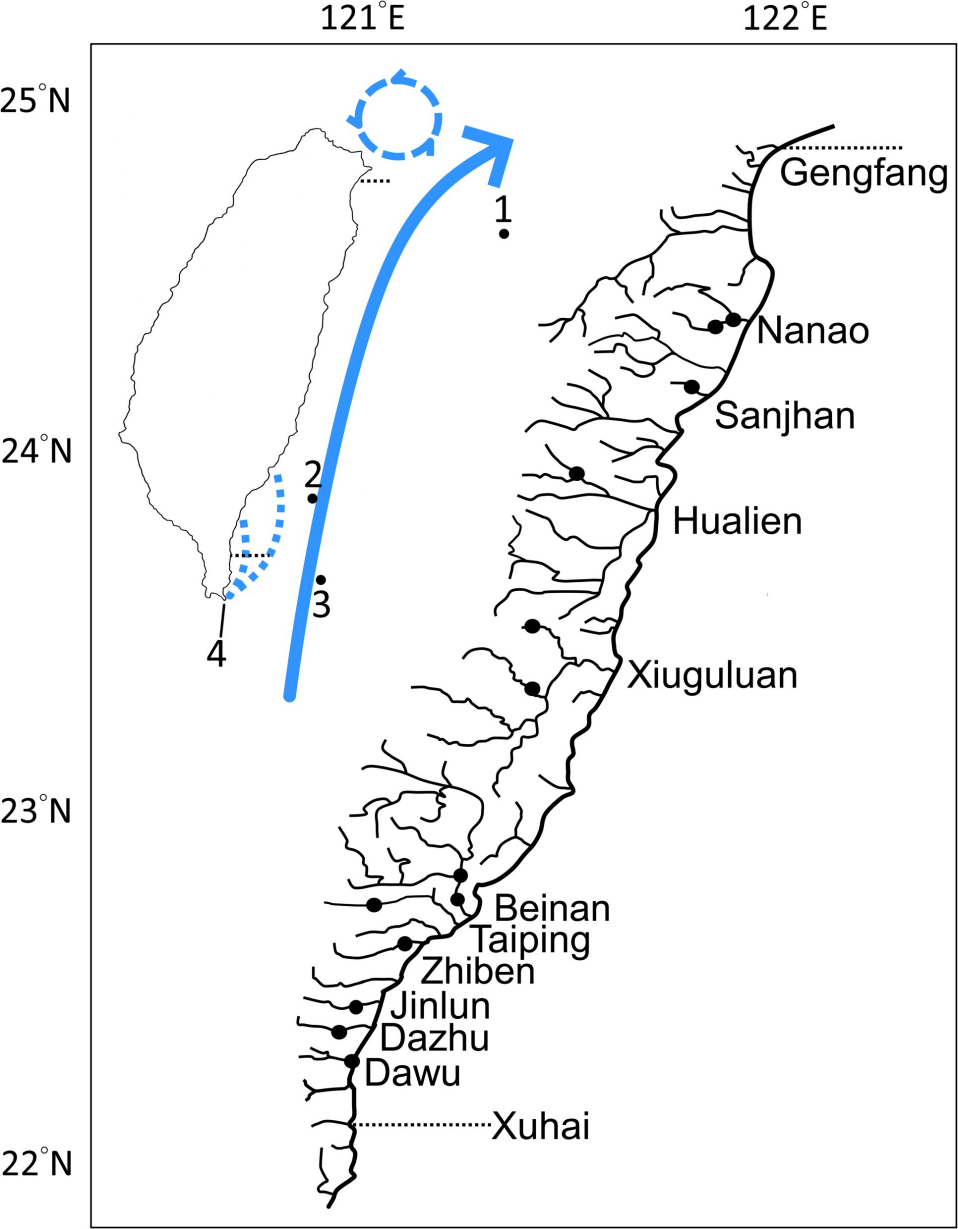
Sampling localities of *Rhinogobius gigas* in Taiwan. The blue dashed circle marks a counterclockwise eddy in the northeastern coasts of Taiwan. Black dots represent approximate sampling sites along the east coast of Taiwan. Sample sizes are given in Table 1. 1 for Yonaguni; 2 for Green Island; 3 for Orchid Island; 4 for the southernmost cape of Taiwan where cyclonic eddies (blue dotted lines for the area they appear) are formed after hit by the Kuroshio Current (the blue bold arrow).

*Rhinogobius* is a genus of small gobies distributed in freshwaters of East Asia, including Cambodia, China, Japan, Korea, Laos, Taiwan, Philippines, Russia, Thailand, and Vietnam [11]. Life histories of species in this genus vary, including amphidromous and landlocked [12–15] while some species are considered facultative amphidromous [13,14,16]. There are 10 species of *Rhinogobius* in Taiwan, including *R*. *candidianus*, *R*. *delicatus*, *R*. *formosanus*, *R*. *gigas*, *R*. *henchuenensis*, *R*. *lanyuensis*, *R*. *maculafasciatus*, *R*. *nantaiensis*, *R*. *rubromaculatus*, and *R*. *similis* [17]. Among these species, *R*. *delicatus*, *R*. *gigas* and *R*. *similis* are the only three rhinogobies native to eastern drainages, but recently *R*. *candidianus* has been introduced from western Taiwan and becoming dominant. *Rhinogobius delicatus* inhabits in upper reach while *R*. *gigas* and *R*. *similis* are commonly found in middle and lower reaches. *Rhinogobius gigas* is endemic to Taiwan, distributed from the Xuhai Creek to Gengfang Creek in Taiwan, but rarely seen in the north of the Nan-ao Creek (Fig 1). This species is one of the largest rhinogoby in Taiwan diagnosed by two or three reddish stripes near pectoral base, more longitudinal scale rows, more pectoral fin rays, and sexually dimorphic with an enlarged cheek and mouth in adult males. *Rhinogobius gigas* is an amphidromous fish and amphidromy is a diadromous behavior that may facilitate distant dispersal due to planktonic larvae transported by oceanic currents when living in the estuary or sea. Fishes with the diadromous behavior are expected to have a broader distribution range [1], although more factors may be involved in shaping their distribution [6–8]. However, some exceptions are observed [4] and *R*. *gigas* is an additional exception with a limited distribution range. *Rhinogobius gigas* has been documented to be retained inshore near the river plume based on lower otolith Sr/Ca ratio with a marine larval stage of 30–40 days after hatching [13]. The short larval stage and local retention near the river plumes may account for the endemism of *R*. *gigas*.

The endemic distribution and short marine larval stage of *R*. *gigas* may imply a low possibility being transported distantly by oceanic currents and this may further imply low connectivity among populations, and moreover, population genetic structure. The aim of the present study is to test whether the population genetic structure is present due to the short marine larval stage of *R*. *gigas*. To test the population connectivity, fragments of mitochondrial displacement loop (D-loop) and cytochrome c oxidase subunit I (COI) were sequenced to provide molecular inference for genetic structure among populations. The results will provide inferences for the influence of Kuroshio Current on the phylogeography of amphidromous fishes in eastern Taiwan.

## Material and methods

### Ethics statement

All sampling sites in the present study were not within any protected area and no permission was required for sampling. Specimen collections were performed in strict accordance with the Wildlife Conservation Act in Taiwan. No ethical approval was required for this study because *R*. *gigas* was not an endangered species protected by the Wildlife Conservation Act in Taiwan and no experiment was conducted on live individuals.

### Samplings

The distribution of *R*. *gigas* is eastern Taiwan with occasional records in the north of Nan-ao Creek and, therefore, not included in the analyses of the present study. A total of 191 individuals of *R*. *gigas* were sampled from 10 basins of eastern Taiwan, including Nanao (NA), Sanjhan (SJ), Hualien (HL), Xiuguluan (XL), Beinan (BN), Zhiben (ZB), Taiping (TP), Jinlun (JL), Dazhu (DZ), Dawu (DW) (Fig 1, and Table 1). Most of the samples were collected by a hand net or electro-fishing (permit number: 1091248447), and sacrificed by immersion in ice water immediately after capture. All specimens were preserved in 95% ethanol, cataloged and deposited in the collection of the Department of Oceanography, National Sun Yat–sen University (DOS), Kaohsiung.

**Table 1:** Sampling sites and diversity indices.

Basin	Code	N	COI			D-loop			concatenated		
			N allele	*h*	*π*	N allele	*h*	*π*	N allele	*h*	*π*
Nanao River	NA	26	3	0.151 ± 0.093	0.0003 ± 0.0002	12	0.883 ± 0.046	0.0035 ± 0.0004	14	0.908 ± 0.040	0.0018 ± 0.0002
Sanjhan River	SJ	10	1	0.000 ± 0.000	0.0000 ± 0.0000	9	0.978 ± 0.054	0.0051 ± 0.0008	9	0.978 ± 0.054	0.0026 ± 0.0004
Hualien River	HL	29	4	0.200 ± 0.098	0.0004 ± 0.0002	12	0.889 ± 0.035	0.0032 ± 0.0004	14	0.916 ± 0.029	0.0017 ± 0.0002
Xiuguluan River	XL	28	4	0.206 ± 0.100	0.0004 ± 0.0002	12	0.886 ± 0.037	0.0034 ± 0.0005	15	0.915 ± 0.036	0.0018 ± 0.0002
Beinan River	BN	28	2	0.071 ± 0.065	0.0001 ± 0.0001	15	0.926 ± 0.030	0.0039 ± 0.0005	15	0.926 ± 0.030	0.0018 ± 0.0003
Zhiben River	ZB	19	1	0.000 ± 0.000	0.0000 ± 0.0000	11	0.930 ± 0.036	0.0048 ± 0.0006	11	0.930 ± 0.036	0.0023 ± 0.0003
Taiping River	TP	1	1	N/A	N/A	1	N/A	N/A	1	N/A	N/A
Jinlun River	JL	32	3	0.123 ± 0.078	0.0002 ± 0.0001	11	0.810 ± 0.059	0.0030 ± 0.0010	13	0.837 ± 0.055	0.0016 ± 0.0002
Dazhu River	DZ	17	2	0.118 ± 0.101	0.0004 ± 0.0004	11	0.941 ± 0.036	0.0050 ± 0.0008	12	0.949 ± 0.037	0.0026 ± 0.0004
Dawu River	DW	1	1	N/A	N/A	1	N/A	N/A	1	N/A	N/A
	total	191	13	0.562 ± 0.017	0.0011 ± 0.0001	45	0.892 ± 0.013	0.0038 ± 0.0002	69	0.956 ± 0.006	0.0024 ± 0.0001

Based on COI (554 bp) and D-loop (520 bp) sequences of 10 populations of *Rhinogobius gigas* from Taiwan. N for sample size; *h* for haplotype diversity; π for nucleotide diversity.

### Molecular analyses

The genomic DNA was extracted from the tissues samples of our collections using the TOOLS EasyPrep HY Tissue & Cell Genomic DNA Extraction Kit (BioTOOLS, Taipei, Taiwan) following the manufacturer’s instructions. Fragments of mitochondrial D-loop and COI were amplified by polymerase chain reaction (PCR) with universal primers, PK2 (5’-GTCGA CTCTC ACCCC TGGCT CCCAA AG-3’) and PU2 (5’-GGGCA TTCTC ACGGG GATGC G-3’; [18]) for D-loop and FishF1 (5’-TCAACCAACCACAAAGACATTGGCAC-3’) and FishR2 (5’-ACTTCAGGGTGACCGAAGAATCAGAA-3’; [19]) for COI.

PCR reactions were run in a 25 μl total volume including 2 μl DNA sample, 1.2 μl for each primer, 3 μl Pro Taq 10X Buffer (Protech, Taipei, Taiwan), 2 μl dNTP (Protech, Taipei, Taiwan), 0.5 μl Pro Taq Plus (Protech, Taipei, Taiwan) and 15.1 μl ultrapure water. Different thermal regimes were applied for the two genes. The thermal regime for D-loop were: an initial denaturation step preheating at 94°**C** for 5 minutes; 35 cycles of 94°**C** for 60 seconds for DNA denaturation, 50°**C** for 30 seconds for primers annealing and 72°**C** for 90 seconds for sequences extension; and a final extension step at 72°**C** for 7 minutes. The thermal regime for COI included an initial denaturation step preheating at 94°**C** for 5 minutes, followed by 37 cycles of 94°**C** for 60 seconds for DNA denaturation, 60°**C** for 45 seconds for primers annealing and 72°**C** for 60 seconds for sequences extension, and a final extension step at 72°**C** for 10 minutes. After checking qualities by electrophoresis, PCR products were purified using the SAP–Exo purification kit (Jena Bioscience, Jena, Germany) according to the manufacturer’s protocols and then sent for sequencing by a commercial company.

All sequencing results were corrected and edited manually using MEGA X [20], and blasted in the database on NCBI (National Center for Biotechnology Information). Sequences of COI and D-loop were concatenated for the following analyses. All sequences used in this study were submitted to the GenBank online database (accession numbers, MT488425–MT488435 and MW018413–MT018414 for COI; MT497480–MT497507 and MW024771–MW024787 for D-loop). Estimated DNA sequence variation within and between populations was computed by DNA Sequence Polymorphism version 6.12.03 software (DnaSP; [21]) for the haplotype number (Nh), haplotype diversity (h) and nucleotide diversity (π).

A maximum likelihood (ML) tree was reconstructed using MEGA version X based on Tamura-Nei (TN93) model with combination gamma distribute with invariant sites (G+I), selected by the lowest bias–corrected Akaike Information Criterion (AICc) value in the model test, and a 1,000 replicate bootstrap probability was analyzed [22]. Sequences of *R*. *formosanus* (accession numbers: KU944938 for COI; EU352724 for D-loop) were used as outgroup. Median joining network (MJN) [23] was implemented in PopART v.1.7 software [24] to deduce the relationships among haplotypes.

To test the historical demography, mismatch distribution and neutrality tests including Tajima’s *D* and Fu’s *Fs* were analyzed using Arlequin ver. 3.5.2 [25]. The Bayesian skyline plot and the most recent common ancestor (TMRCA) were estimated for all specimens only based on COI sequences due to the unavailability of substitution rate for D-loop. Both analyses were conducted using the software BEAST version 1.7.5 [26], calculated with Markoc Chain Monte Carlo (MCMC) and TN93 + G + I model, run for 10 million generations with sampling every 1,000 generations and 10% burn-in of the samples. The substitution rate of 1.9% per million years (My) of COI gene in species of the Gobiidae was applied according to Keith et al. [8]. In the TMRCA analysis, the effective sample size values for each parameter exceeded 200, and the 95% highest posterior density (HPD) intervals were reported. The results of Bayesian skyline plot and TMRCA was read in TRACER ver. 1.7.4 [27].

## Results

### Genetic diversity

A 1,074-bp concatenated sequence of mtDNA COI (554 bps) and D-loop (520 bps) was analyzed for 191 individuals obtained from 10 sampling sites (Fig 1) in rivers of eastern Taiwan. In total, 13 variable sites and 10 parsimoniously informative sites were observed. Among the 191 concatenated sequenced, 69 unique haplotypes were identified. One sequence of each haplotype of COI and D-loop were respectively submitted to GenBank. The 69 haplotypes of concatenated sequences were summarized in S1 Table with duplicated number of each haplotype. The mean haplotype diversity and nucleotide diversity for all samples were 0.956 and 0.0024, respectively. Thirteen haplotypes of COI gene were recognized with the mean of haplotype and nucleotide diversities for all samples 0.562 and 0.0011, respectively. Haplotype diversity of each population was very low, ranging from 0 (ZB and SJ) to 0.206 (XL). In D-loop, 45 haplotypes were identified with the means of haplotype and nucleotide diversities for all samples of 0.892 and 0.0038, respectively. The haplotype diversity of each population ranges from 0.810 (JL) to 0.978 (SJ). The nucleotide diversity of all samples was fairly low, ranging from 0.0030 (JL) to 0.0051 (SJ). Haplotypes diversity and nucleotide diversity of each population were summarized in Table 1.

### Molecular analyses

**Fig 2:**
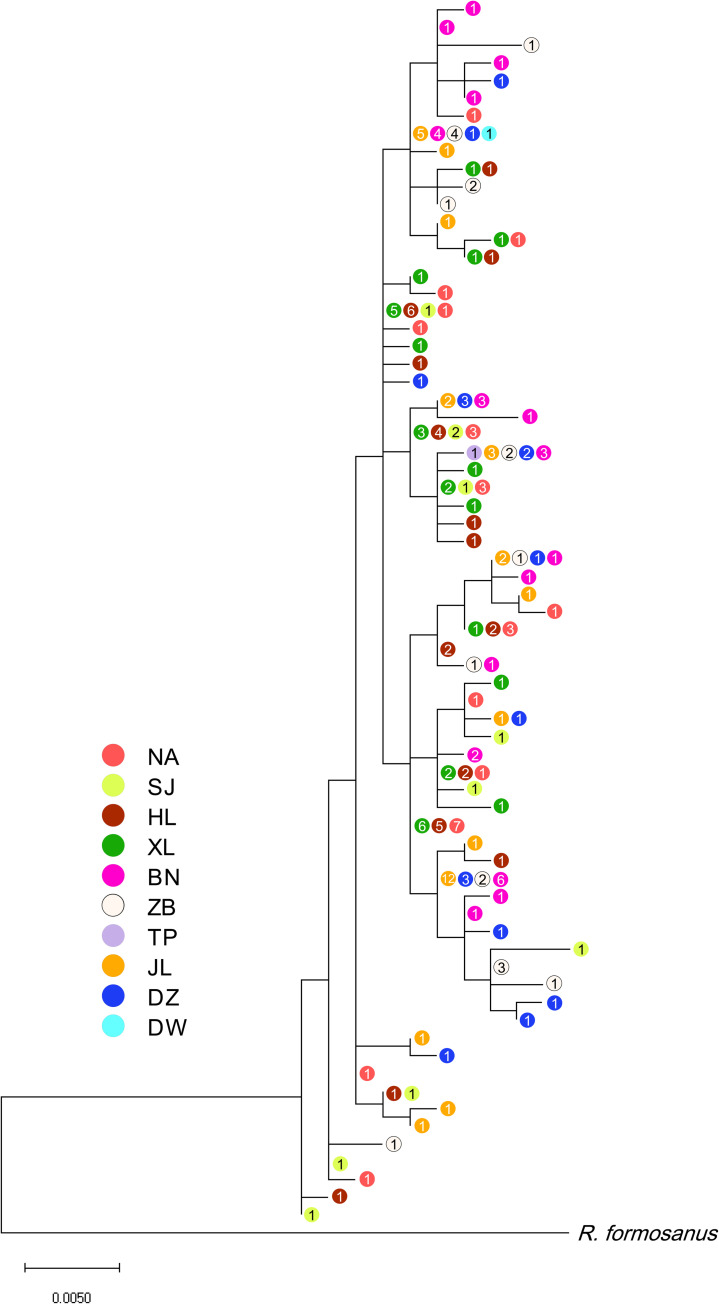
The maximum likelihood tree for *Rhinogobius gigas*. Based on haplotypes of 191 concatenated mtDNA sequences (1074 bps). Bootstrap values of all nodes are lower than 55 and not shown. Colors represent different sampling basin; numerals within circles represent number of individuals; basin names of codes are given in Table 1.

**Fig 3:**
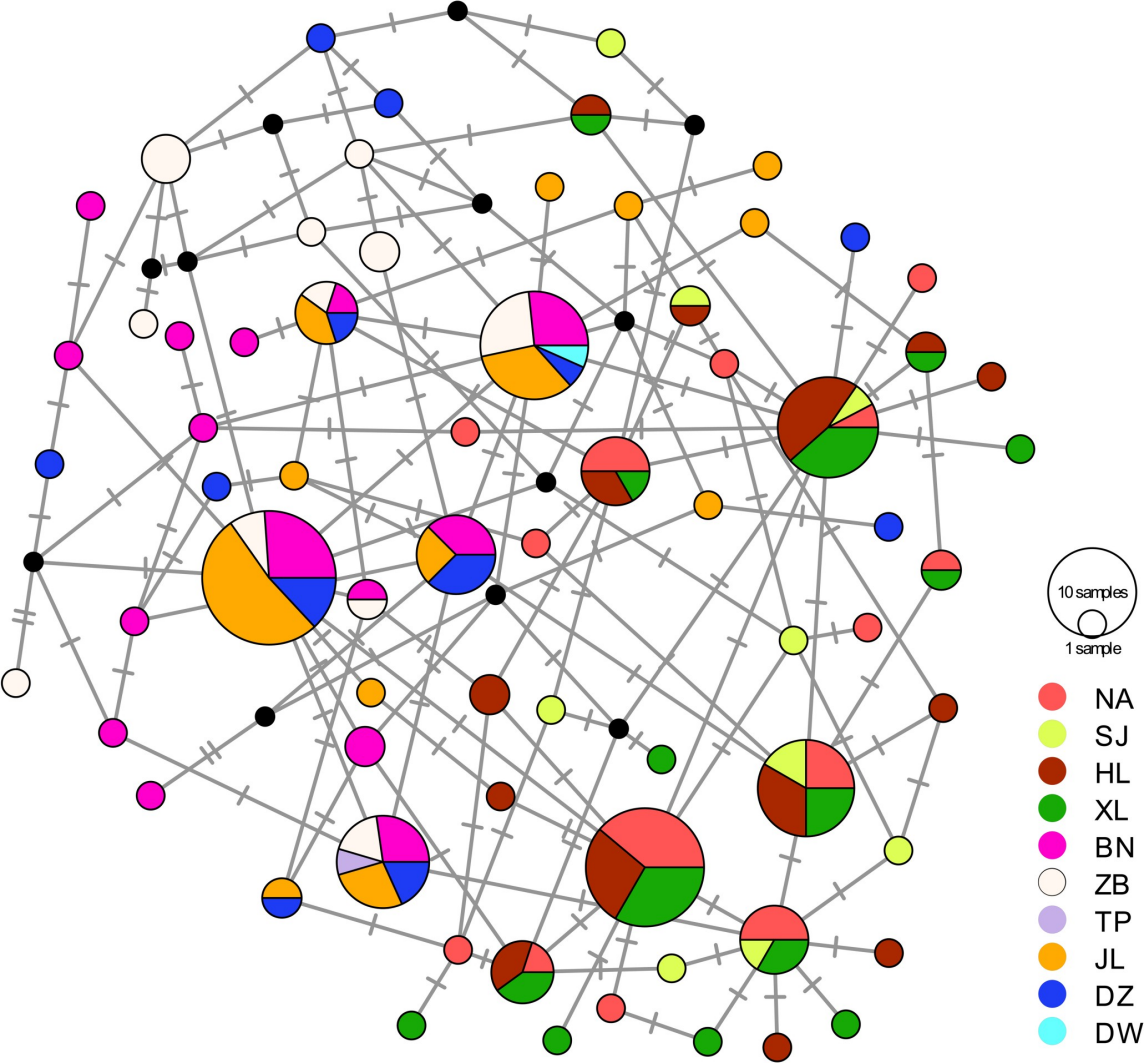
Median-joining network of 191 concatenated mtDNA sequences (1074 bps) of *Rhinogobius gigas*. Colors represent different sampling basins; size of pie charts is proportional to the number of individuals; dashes and black circles are haplotypes not collected in this study; basin names of codes are given in Table 1.

**Fig 4:**
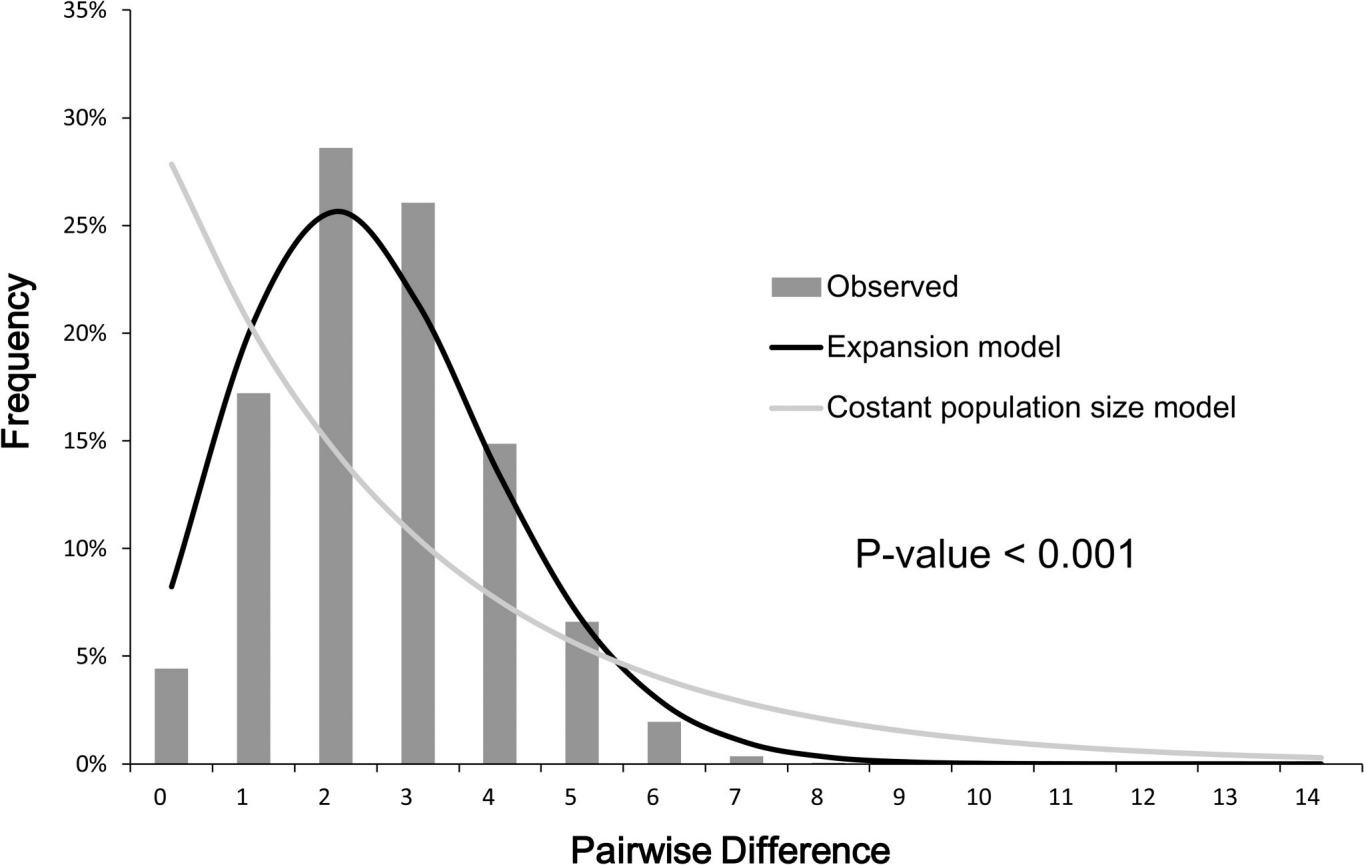
Mismatch distributions of concatenated mtDNA (1074 bp) of *Rhinogobius gigas*. The black line is the expected distribution calculated for the assumption of a demographically expanding population; gray line is the expected distribution under the constant population model; gray bars indicate the observed frequencies of pairwise differences of nucleotides among haplotypes.

**Fig 5:**
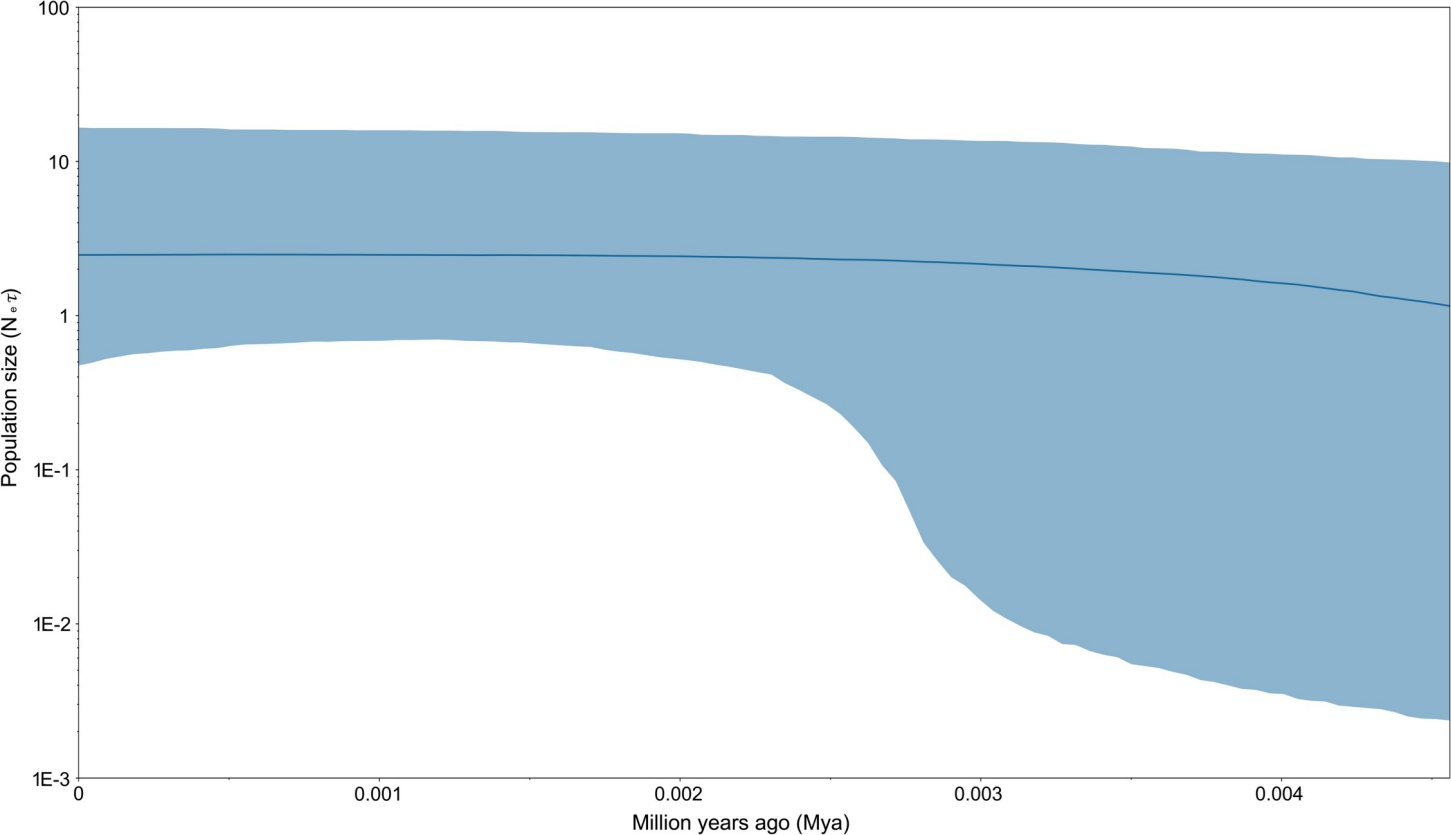
Bayesian skyline. Bayesian skyline plot of changes of effective population size through time for *Rhinogobius gigas*.

The phylogenetic analysis revealed no genetic structure among the 191 individuals and no long internal branch was found. The bush-like MJN without predominantly shared haplotypes support the general topology of the ML tree (Figs 2 and 3). The mismatch distribution of concatenated sequences of COI and D-loop was shown in Fig 4. A left-hand side unimodal pattern suggested the population may have undergone a sudden expansion. The population expansion was further supported by the significantly negative values of Fu’s *Fs* (-26.6; *p* < 0.001) and Tajima’s *D* (-1.5; *p* = 0.037) values, which failed to reject the null hypothesis of constant population size. According to the Bayesian skyline analysis, all populations of *R*. *gigas* had a stable growth rate, not showing significant population dynamics during the past five thousand years (Fig 5). The TMRCA of all COI sequences of *R*. *gigas* were dated to 0.13 mya (effective sampling size, 3578.5; 95% HPD, 0.04–0.24 mya).

## Discussion

### Genetic diversity and population demography

According to the results (Table 1), the analysis of genetic diversity of D-loop showed high haplotype diversity and low nucleotide diversity with the mean values of 0.892 ± 0.013 and 0.0038 ± 0.0002, respectively. The pattern of high haplotype and low nucleotide diversities of D-loop is similar to the congener *Rhinogobius maculafasciatus* [28], amphidromous gobies of a different subfamily [29–31] and a cyprinid in eastern Taiwan, *Aphyocypris kikuchii* [32]. In COI gene, unlike the pattern of D-loop, both the haplotype (0.562 ± 0.017) and nucleotide diversities (0.0011 ± 0.0001) are rather low. Despite that high COI haplotype diversity was observed in other gobies [30], the low haplotype diversity of COI suggests that this gene may not be a good marker for population genetic study on species of the genus *Rhinogobius*. Therefore, we included D-loop gene, which provided more genetic variation and higher resolution of the genetic structure in this study.

High haplotype diversity but low nucleotide diversity of concatenated sequences implies a bottleneck followed by a recent population expansion. Mismatch distribution and negative values of Fu’s *Fs* and Tajima’s *D* also support a demographic expansion that may be attributed to the postglacial warmer climate commonly observed in fishes [33]. The Bayesian skyline analysis showed insignificant population dynamics during the past five thousand years. The recent stable population dynamics implies the expansion scenario probably occurred after the last glacial, but before the insignificant population dynamics. However, the incongruence between mismatch distribution and the Bayesian skyline analysis could be a consequence of different datasets (COI + D-loop vs. COI, respectively). We analyzed mismatch distribution, Fu’s *Fs* and Tajima’s *D* based on COI and the significant demographic expansion was also retrieved (data not shown). Therefore, the different datasets do not account for the incongruence. The TMRCA of *R*. *gigas* were dated to 0.13 mya, approximately at the beginning of the last glacial (0.128 to 0.116 mya; [34]). The sea level changes may provide the opportunity for allopatric speciation [35].

### Genetic structure and distribution

Previous studies have shown that there is no genetic structure among populations of freshwater fishes in eastern Taiwan, including four primary freshwater fishes, *A*. *kikuchii* (except for one population), *Hemimyzon taitungensisi*, *Onychostoma alticorpus* and *Spinibarbus hollandi* [18,32,36] and one amphidromous fish *Sicyopterus japonicus* [29]. The former four are primary freshwater fishes intolerable to salt water so their genetic homogeneity is possibly due to head-water capture [18,32] while that of the amphidromous *S*. *japonicus* may result from frequent gene flow through pelagic larvae dispersion in the ocean [29]. Similar to the results of previous studies, the ML tree shows that none of the *R*. *gigas* population is monophyletic (Fig 2) and the bush-like pattern of the MJN (Fig 3) implies lack of correlation between sampling sites and genetic variation. Phylogenetic analyses show apparent absence of population genetic structure in the amphidromous *R*. *gigas* endemic to eastern Taiwan. The inter-population genetic homogeneity and intra-population genetic variability of *R*. *gigas* (Table 1; Figs 2 and 3) may have arisen from frequent gene flow through passive transportation during PLD as observed in *S*. *japonicus* [29,31] and *Stiphodon percnopterygionus* [30].

Freshwater species with a diadromous life history may exhibit higher dispersal ability [30,37]. *Sicyopterus japonicus* and *S*. *lagocephalus* with long PLD for 130 to 198 days and 133 to 266 days, respectively [38,39] are broadly distributed across ranges of c. 2400 and 18000 km, respectively [1]. On the other hand, amphidromous species, such as *S*. *aiensis* and *S*. *sarasini*, with shorter PLD of c. 80 days are considered endemic to Vanuatu and New Caledonia, respectively [4], separated by a straight line distance of approximately 400 km. However, PLD of the amphidromous *R*. *gigas* is around 30–40 days [13], which is very short compared to the aforementioned gobies and an amphidromous congener, *R*. *formosanus*, with a PLD of 38 to 89 days (mean = 58.8), and similar to the landlocked population of *R*. *formosanus* with a PLD of 24 to 46 days (mean = 38.8; [16]). The short PLD of *R*. *gigas* may constrain the larval dispersal and account for the endemism as proposed by Shiao et al [13].

The strong Kuroshio Current plays an important role in facilitating wide distribution of many marine lives along the northwestern Pacific Ocean [40]. Based on the current speed of 100–150 cm s^-1^ [41,42], the Kuroshio Current can transport larval fish across more than 1000 km in a month. Since *R*. *gigas* is endemic to eastern Taiwan, it is unlikely for *R*. *gigas* larvae to disperse by the Kuroshio Current and this may be further supported by its absence from the Green Island (approximately 33 km away from eastern Taiwan), Orchid Island (73 km away) and Yonaguni (110 km away) along the Kuroshio Current. Therefore, we hypothesize that larvae of *R*. *gigas* may actively select the habitat close to estuaries and avoid to be entrained in the Kuroshio Current, which is approximately several kilometers away from the eastern coasts of Taiwan. Active habitat selection by pre-settlement larvae has been documented in many reef fishes [43] and some amphidromous gobies. For example, *Sicydium punctatum* actively choose low to intermediate salinity at 5-d post-hatch and minimized exposure to salinities >10 ppt [44].

Disregarding distant dispersal, *R*. *gigas* may expand its’ distribution via the tidal current dispersal to neighboring rivers in eastern Taiwan and this may result in gene flow accounting for the genetic homogeneity observed in the present study. However, *R*. *gigas* is endemic to eastern Taiwan with very rare reports in the distal ends of distribution, the Gengfang Creek and Lanyang River in the north and the Xuhai Creek in the south (Fig 1). The northward dispersal may be constrained by a counterclockwise eddy that results in southward coastal currents in the northeastern coasts of Taiwan [45] (Fig 1). In addition, inter-specific competition may also be a constraint on the distribution of *R*. *gigas*. *Rhinogobius gigas* is syntopic with *R*. *formosanus* in the Gengfang Creeks and with *R*. *maculafasciatus* in the Lanyang River in northeastern Taiwan (Fig 1). In the syntopic rivers, *R*. *maculafasciatus* and *R*. *formosanus* are absolutely dominant in terms of numbers. The northward range expansion of *R*. *gigas* may be confined by interspecific competition with its congeners. Similarly, in southeastern Taiwan, cyclonic eddies are formed at the southernmost cape of Taiwan by the hit of strong Kuroshio Current [46] (Fig 1). The complicated eddies may restrain the southward dispersal. However, passive transportation of larvae by oceanic currents is complicated. Spawning season, length of PLD, characteristics of planktonic environments, seasonal variation of currents and other biological and physical factors may influence the dispersal [47]. More studies, such as comparative phylogeography, are needed to answer the limited distribution of this species.

## Conclusion

In the present study, mitochondrial concatenated sequences of 191 *R*. *gigas* from 10 populations show high haplotype diversity and low nucleotide diversity. Combined with the left-hand side unimodal pattern of mismatch distribution and negative values of Fu’s *Fs* and Tajima’s *D*, our results imply a demographic scenario of a bottleneck followed by a recent population expansion. The genetic homogeneity is probably due to the amphidromous life history providing the opportunity for passive egg and/or larval transportation among the rivers in eastern Taiwan. The endemism to eastern Taiwan may be a consequence of complicated interactions among short PLD, active habitat selection by larvae, interspecific competition and coastal currents. Further studies such as comparative phylogeography may be needed to uncover the complicity.

## Supporting information

S1 Table Accession-number combinations of haplotypes of concatenated sequences. Numbers in parenthesis are duplicated copies of each haplotype.
(DOCX)
