# Supplementary material for: Amphidromous but endemic: Population connectivity of Rhinogobius gigas (Teleostei: Gobioidei)
Source: PLoS One. 2021 Feb 11;16(2):e0246406. doi: 10.1371/journal.pone.0246406 (PMC7877787; doi:10.1371/journal.pone.0246406)
Supplement: S1 Table — Numbers in parenthesis are duplicated copies of each haplotype. (DOCX) [file pone.0246406.s001.docx]

**Supporting information**

**S1 Table. Accession-number combinations of haplotypes of concatenated sequences.** Numbers in parenthesis are duplicated copies of each haplotype.
